# Non-Destructive Identification of Naturally Aged Alfalfa Seeds via Multispectral Imaging Analysis

**DOI:** 10.3390/s21175804

**Published:** 2021-08-28

**Authors:** Xuemeng Wang, Han Zhang, Rui Song, Xin He, Peisheng Mao, Shangang Jia

**Affiliations:** College of Grassland Science and Technology, China Agricultural University, Beijing 100193, China; xuemeng.wang@cau.edu.cn (X.W.); hanzhang003@cau.edu.cn (H.Z.); ruisong2021@cau.edu.cn (R.S.); xin.he@cau.edu.cn (X.H.); maops@cau.edu.cn (P.M.)

**Keywords:** aged seeds, multispectral imaging, multivariate analysis, alfalfa, non-destructive identification

## Abstract

Seed aging detection and viable seed prediction are of great significance in alfalfa seed production, but traditional methods are disposable and destructive. Therefore, the establishment of a rapid and non-destructive seed screening method is necessary in seed industry and research. In this study, we used multispectral imaging technology to collect morphological features and spectral traits of aging alfalfa seeds with different storage years. Then, we employed five multivariate analysis methods, i.e., principal component analysis (PCA), linear discrimination analysis (LDA), support vector machines (SVM), random forest (RF) and normalized canonical discriminant analysis (nCDA) to predict aged and viable seeds. The results revealed that the mean light reflectance was significantly different at 450~690 nm between non-aged and aged seeds. LDA model held high accuracy (99.8~100.0%) in distinguishing aged seeds from non-aged seeds, higher than those of SVM (87.4~99.3%) and RF (84.6~99.3%). Furthermore, dead seeds could be distinguished from the aged seeds, with accuracies of 69.7%, 72.0% and 97.6% in RF, SVM and LDA, respectively. The accuracy of nCDA in predicting the germination of aged seeds ranged from 75.0% to 100.0%. In summary, we described a nondestructive, rapid and high-throughput approach to screen aged seeds with various viabilities in alfalfa.

## 1. Introduction

Seed aging is an irreversible and natural process in which the vigor of seeds declines or loses completely. Seed aging has been a popular issue in seed research, as aged seeds lower seed emergence and growth, reduce overall germination performance and limit seed production [1,2,3]. Therefore, it is of great significance to distinguish aged seeds for ensuring seed quality and reducing economic losses. The traditional inspection of aged seeds is based on the indicators of color and aroma, germination test, tetrazole staining [4,5,6], etc. However, all these methods are time-consuming, and the seed cannot maintain its original state, including when being discarded [7,8]. In recent years, spectroscopic techniques have shown great potential in detecting the seeds. For example, Fourier spectroscopy and near-infrared spectroscopy (NIRS) have been used to examine seed vitality [9,10,11,12]. Tigabu and Oden [13] applied NIRS into the detection of viability detection of Masson pine seeds and found that the seeds with different aging times (3 d, 7 d, and 9 d) were identified with an accuracy of 80%. Yang et al. [14] utilized NIRS and BP neural network to classify artificially aged maize seeds into three degrees with an accuracy of 85%. Although NIRS is an optical spectroscopy method that successfully noninvasively characterizes seeds, it only employs infrared light with 1000–2500 nm wavelength range and cannot cover spectral data similar to other spectral techniques, such as hyperspectral imaging (HSI) and multispectral imaging (MSI) [15]. Based on hundreds of thousands of bands, HSI was successfully applied in seed quality and safety inspection in maize, soybean, and wheat [16,17,18]. 

MSI is an emerging analytical and detection technology, which integrates conventional imaging and spectroscopy to simultaneously attain both spatial and spectral information. It is simple, practical and non-destructive with low cost. Currently, MSI has been widely used in different types of identification and quality testing, such as cultivar and variety discrimination of seeds in alfalfa and rice [19,20], seed vigor and vitality detection in castor and soybean [21,22], and seed health screen in cowpea and wheat [23,24]. According to the physical and chemical difference between aged seeds and non-aged seeds [25,26], MSI may have a great potential in distinguishing aged seeds from non-aged seeds. However, MSI mainly was applied in the studies on crop seeds [22,27] rather than on forage seeds. Forage seeds are smaller than crop seeds in size; thus, they are more difficult to distinguish for seed characteristics. Alfalfa (*Medicago sativa*) seed is one of the most important forage seeds. Based on the production data of forage seeds in 2017, the total yield of alfalfa seed reached 12,217 t in China and 28,980 t in the U.S. [28,29], which is higher than those of other forage seeds. In addition, a large amount of alfalfa seeds is imported from abroad to China each year [30]. The giant quantity of alfalfa seeds in production and trade brings great pressure to the storage, which results in the phenomenon of seed aging inevitably. Therefore, it is necessary to distinguish the alfalfa seeds with different qualities, such as storage of various years, and a fast and non-destructive identification method is useful in both application in trade and planting and research in alfalfa aged seeds. In this study, we collected the multispectral information of naturally aging alfalfa seeds, which were stored in our lab for up to 15 years, and employed five multivariate analysis methods to classify aged alfalfa seeds and predict their germination. We proposed a fast and non-destructive identification method with high accuracies in an attempt to provide guidance for the classification and detection of aging seeds in both seed research and field sowing.

## 2. Materials and Methods

### 2.1. Seed Sample

“Zhongmu NO.2” seeds under 1~15 storage years were provided by the Official Herbage and Turfgrass Seeds Testing Center, Ministry of Agriculture and Rural Affairs, China (Supplementary Figure S1). Seeds harvested in 2004, 2008, 2011, 2017 and 2019 were kept in valve-bags in the laboratory conditions (25 °C, 35% relative humidity) until the time of image acquisition in November 2020. For each year, 100 seeds were used for the experiments. We defined the seeds harvested in 2019 as non-aged seeds (CK). The other seeds of different naturally aging years were classified into aged seeds. Meanwhile, in order to further test the ability of multispectral data in predicting seed germination, the seeds in the five groups (2004, 2008, 2011, 2017, 2019) were divided into two groups: germinating seeds (G) and non-germinating seeds (NG).

### 2.2. Germination Test

Germination percentage and potential were surveyed to reflect seed vigor. We collected multispectral information of all the seeds before germination test, and 25 seeds were placed on a wet pleated filter paper in one petri dish with a diameter of 11 cm. Germination was performed by rules of “seed testing for forge, turfgrass and other herbaceous plant” (GB/T 2930.4-2017), and counting seeds was performed every day to calculate seed germination percentage and germination potential as followed.

Germination percentage (%) = (no. of seeds germinated at the end time/no. of all seeds) × 100(1)

Germination potential (%) = (no. of seeds germinated at the first time/no. of all seeds) × 100(2)

### 2.3. MSI Data Recording

The multispectral imaging data was recorded in MSI VideometerLab4 (Videometer A/S, Herlev, Denmark), which contains 19 monochrome light-emitting diodes (LEDs) with non-uniform wavelength distribution, i.e., 375, 405, 430, 450, 470, 505, 525, 570, 590, 630, 645, 660, 700, 780, 850, 870, 890, 910, 940 and 970 nm. The LEDs flash continuously in a scan time of five seconds and produce a monochrome image at each wavelength [31,32]. Before the spectral information was collected, the 100 seeds in the five aging groups were numbered as 1~100, and each petri dish with 25 seeds was placed into the instrument for signal recording. Background information was removed before extracting the spectral information of individual seed. The morphological and spectral features of seeds were exported for further analysis. 

### 2.4. Multivariate Data Analysis

In this study, the morphological and spectral information of seeds collected by MSI was used for multivariate analysis (Supplementary Figure S2), including PCA (principal component analysis), SVM (support vector machine), RF (random forest), LDA (linear discriminant analysis) and nCDA (normalized canonical discriminant analysis). Hold-out method was used for cross validation. 

PCA is a common method for dimensionality reduction analysis of high dimensional data, and as an explorative multivariate data analysis technique, PCA can extract the main features of the data, and has the possibility to distinguish seeds with similar morphological and spectral features.

SVM is often used to solve the problems of data grouping in model recognition, as one of the supervised learning algorithms. The learning strategy of SVM is to maximize inter-class distance and minimize intra-class distance [33]. The number of seeds used for classification was 100 for each sample harvest year. The 70% of the seeds in each sample harvest year were randomly selected as the training set and the other 30% were used for model testing. The classification performance of the model was evaluated by accuracy, sensitivity and specificity [31], with formula as followed.

(3) $$\mathrm{Accuracy}\;(\%)=\frac{\mathrm{TN}+\mathrm{TO}}{\mathrm{Total}}\;\times \;100,$$

(4) $$\mathrm{Sensitivity}\;(\%)=\frac{\mathrm{TN}}{\mathrm{TN}+\mathrm{FO}}\;\times \;100,$$

(5) $$\mathrm{Specificity}\;(\%)=\frac{\mathrm{TO}}{\mathrm{TO}+\mathrm{FN}}\;\times \;100.$$

Here, TN: the non-aged seeds were predicted to be non-aged seeds; TO: the aged seeds were predicted to be aged seeds; FN: the aged seeds were predicted to be non-aged seeds; and FO: the non-aged seeds were predicted to be aged seeds.

LDA is a classical algorithm in model recognition, with the similar calculation to that in SVM. The data dimension is reduced to lower latitudes to maximize inter-class distance and minimize intra-class distance, and the samples receive the best separability in the space. The classification performance of LDA was also evaluated by accuracy, sensitivity and specificity.

RF is a combination classification model based on decision tree predictor, as the classification accuracy of decision tree is higher than that of one single tree. RF is a natural nonlinear modeling method and has a high prediction accuracy.

The nCDA method is employed as a supervised transformation construction method to divide the images into regions of interest with different spectral characteristics [26]. nCDA is known as supervised Fishers linear classifier based on MSI transformation of the images, and the learning strategy is to minimize the Jeffries–Matusita distance among the observations within a group and maximize the distance among the known groups [24]. In the seeds with different spectral features, the regions of interest with different spectral strength are marked and colored based on standardization of the spectral information of specified samples [34]. In this study, red and blue colors are used for standardization of MSI images of seeds harvested in 2019 and 2014, respectively, and the other images were transformed accordingly by nCDA function of MSI-Transformation Builder in Videometer software version 4. 

PCA, RF, SVM and LDA analysis was conducted using R packages of *FactoMineR*, *randomForest*, *e1071* and *MASS*, respectively. The parameter of C could balance the penalization of errors [35], and was used as a regularization meta-parameter in SVM analysis. The parameters of ntree with 500 and mtry with 3 were used in RF. 

In addition, other analysis, for example, statistical analysis for morphological data and reflectance and pixel histogram plotting, was performed in both excel files and Videometer software version 4. 

## 3. Results

### 3.1. Germination and Morphologic Features of Aged and Non-Aged Seeds

**Figure 1:**
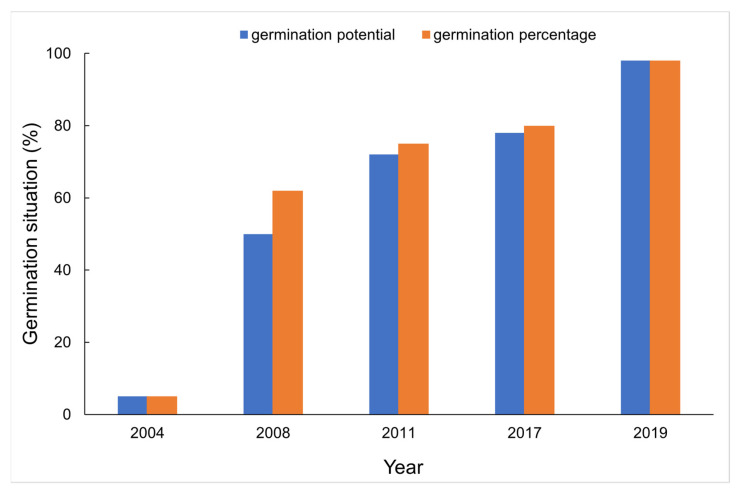
Germination index in naturally aged seeds.

The results of germination test showed that germination percentage of the seeds with four storage years (i.e., harvested in 2004, 2008, 2011 and 2017) was lower than that of the non-aged seeds harvested in 2019, and with the increase of naturally aging years, the germination percentage and germination potential of seeds decreased (Figure 1), especially, compared to the germination percentage of 98% in 2019 in which only 5% was achieved in 2004.

We extracted 14 indexes of morphologic features for the seeds with various naturally aging years (Table 1) and performed the comparative analysis. It showed that, in total, there were 13 morphologic features with significant differences in four comparisons with CK (2004 vs. 2019, 2008 vs. 2019, 2011vs. 2019, 2017 vs. 2019), such as, area, length, width, and hue. Six indexes of area, length, width, CIELab L*, CIELab A* and hue among the four groups were all significantly different from the CK group. The seeds harvested in 2008 held the most differential indexes (13 ones), followed by those in 2017 (12 ones), 2004 (10 ones) and 2011 (7 ones).

### 3.2. Spectroscopic Analysis of Aged Seeds and Non-Aged Seeds

**Figure 2:**
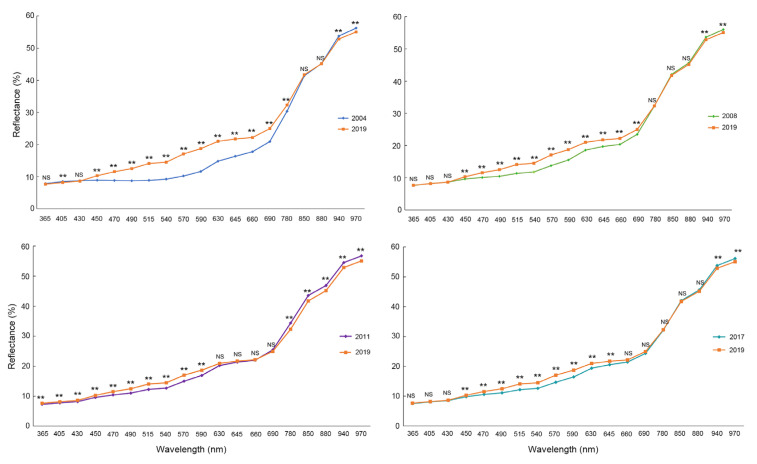
Reflectance of 19 wavelengths in aged seeds vs. non-aged seeds (CK). ** and NS on the lines indicate significant difference and no significant difference, respectively.

Spectral reflectance indicated pixel intensity at 19 wavelengths from the UV (365 nm) to the shortwave NIR (970 nm). We analyzed the spectral reflectance of aged seeds and non-aged seeds under 19 bands and found that they exhibited similar trends (Supplementary Figure S3), especially in the spectral range from 450 nm to 690 nm, CK (harvested in 2019) showed the highest spectral reflectance. In the NIR region (from 850 to 970 nm), this trend disappeared, and the aged seeds harvested in 2011 presented higher reflectance intensities. We explored the four comparisons individually, and the biggest differences were observed between seeds harvested in 2004 and 2019 (Figure 2). All the aged seed groups showed great differences of reflectance intensities from those of non-aged seeds.

**Figure 3:**
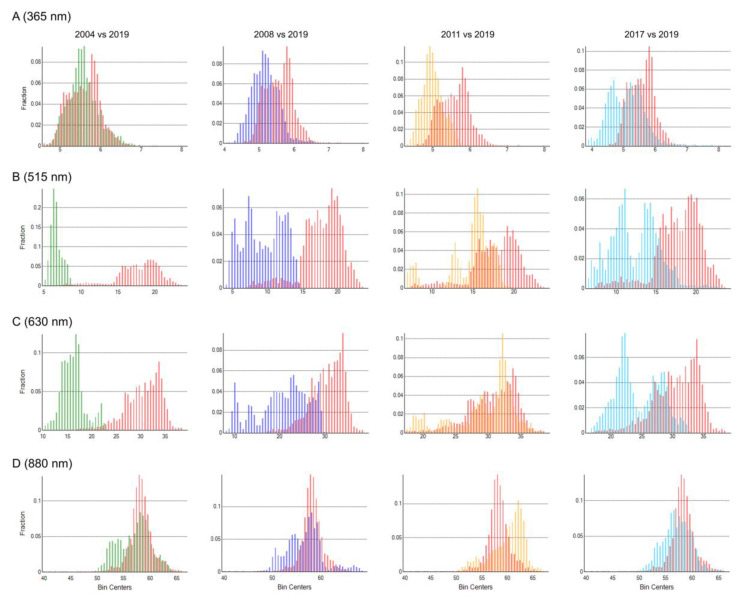
Pixel histogram of aged seeds vs non-aged seeds (CK). (**A**), 365 nm; (**B**), 515 nm; (**C)**, 630 nm; and (**D**), 880 nm. Note: The X-axis of the histogram represents the type of pixels; The Y-axis represents the total number of pixels in each pixel type for each color. Green bars represent seeds harvested in 2004; dark blue bars for seeds harvested in 2008; yellow bars for seeds harvested in 2011; light blue bars for seeds harvested in 2017; and red bars for seeds harvested in 2019.

According to the results of spectral reflectance, we selected two bands in the middle regions (515 nm and 630 nm) and two bands at the two end regions (365 nm and 880 nm) to plot the pixel histogram. The results showed Y-axis pixel values in both 515 nm and 630 nm, which are in the visible light regions (400~760 nm), were significantly different with two separated peaks between aged seeds and CK (Figure 3B,C). There were still differences on distribution peaks between aged seeds and non-aged seeds at 365 nm and 880 nm (Figure 3A,D). The position of the pixel peak of aged seeds moved from the left in Figure 3A to right in Figure 3D.

### 3.3. Multivariate Analysis

**Figure 4:**
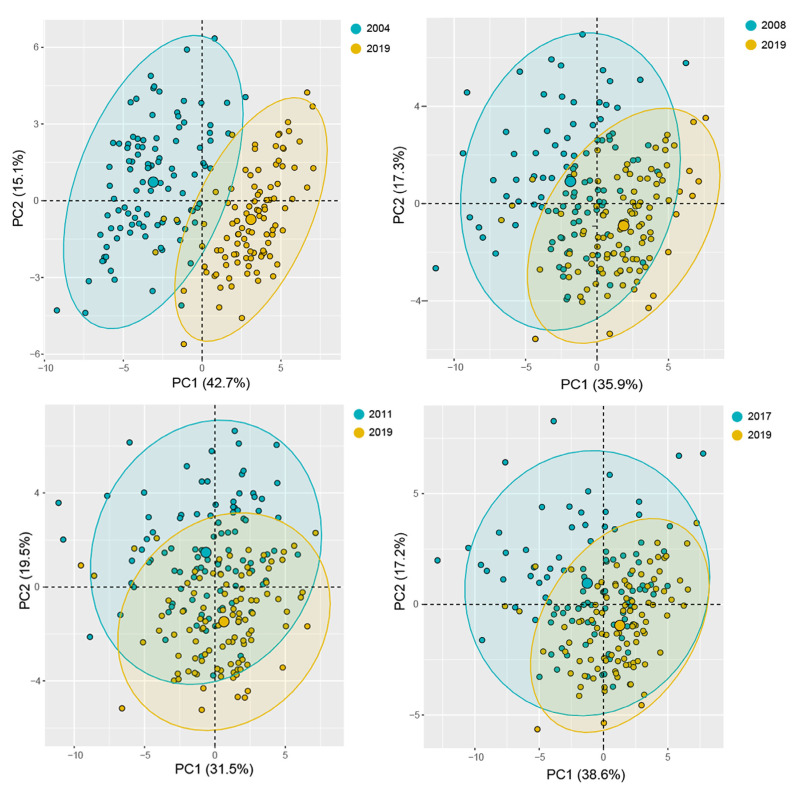
PCA plot based on morphological and multispectral data in seeds aged for various years.

Five multivariate analysis models were developed based on the multispectral data. Firstly, the PCA results based on morphological and spectral features showed that the first two principles components explained 57.8% (2004 vs. 2019), 53.2% (2008 vs. 2019), 51.0% (2011 vs. 2019), 55.8% (2017 vs. 2019) of the original variance. The seeds of 2004 showed the best separation results, and the seeds of 2017 and 2019 could not be distinguished from each other (Figure 4). It is difficult to completely distinguish aged seeds from non-aged seeds by PCA. In contrast, SVM model in classifying aged seeds and non-aged seeds had an average accuracy value as high as 99.3% (2004 vs. 2019), 91.3% (2008 vs. 2019), 90.9% (2011 vs. 2019) and 87.4% (2017 vs. 2019), respectively. The sensitivity and specificity in SVM were also great, with a range from 87.6% to 99.6%, and from 87.3% to 99.0%, respectively. The average accuracy values of RF model in classifying aged seeds and non-aged seeds were 99.3% (2004 vs. 2019), 89.6% (2008 vs. 2019), 85.5% (2011 vs. 2019) and 84.6% (2017 vs. 2019). It was noticed that LDA model exhibited accuracy values as high as 99.8%~100.0%, the best sensitivity with a range from 99.7% to 100.0% and the best specificity of 100% in all the groups (Table 2).

**Figure 5:**
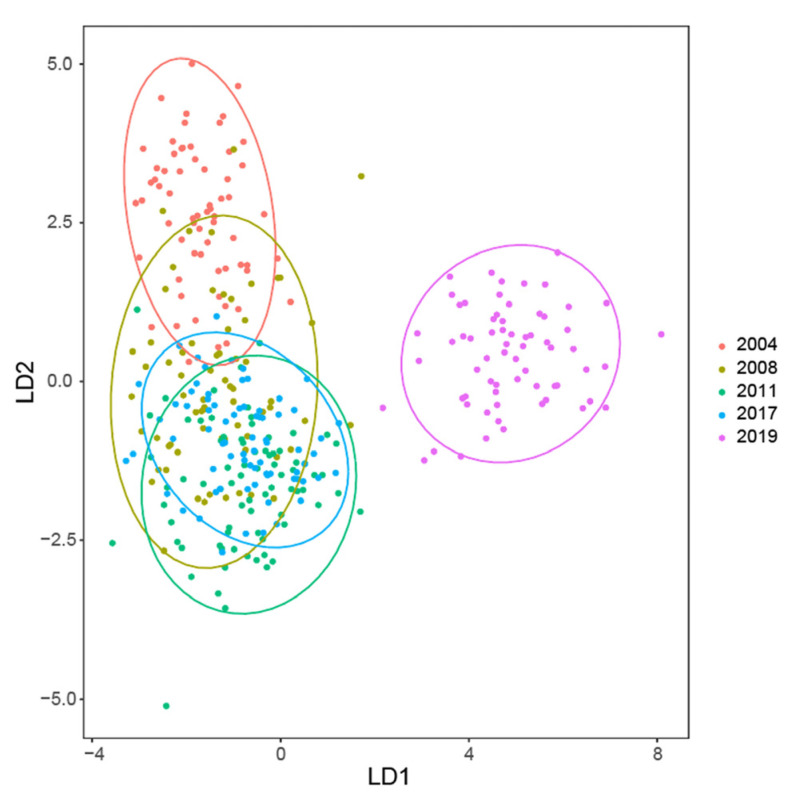
LDA plot based on morphological and multispectral data in aged vs. non-aged seeds.

By combining PCA, SVM, RF and LDA together, we found that LDA model had the best performances on the differentiation and prediction of aged and non-aged seeds. The LDA visualization of five groups of seeds showed the non-aged seeds in 2019 were clearly separated from the other aged seeds (Figure 5), while the seeds in 2004 were also found different from the other aged seed groups. We calculated relative importance of morphological and spectral features for SVM and LDA models and found that spectral features had a great contribution for aged seeds discrimination (Supplementary Figure S4), while in the RF model, CIELab L*, CIELab A* and Hub were major contribution indexes (Supplementary Figure S5). We tested the contributions of morphological and spectral data in three models of SVM, LDA and RF, and found that the combination of two types of features worked with better classification results. For example, “morphological+spectral” data produced 99.8% LDA accuracy in 2017 vs. 2019, and only 98.4% and 79.7% for “morphological only” and “spectral only”, respectively, were obtained (Table 3). Furthermore, we tested LDA performance to predict the storage year, by selecting randomly 90 seeds from each of the five groups (2004, 2008, 2011, 2017, 2019) as the training set and the other 10 seeds as the test set. The average prediction accuracy of storage years ranged from 90% to 98%. 

### 3.4. Multivariate Analysis of Germinated Seeds and Non-Germinated Seeds

**Figure 6:**
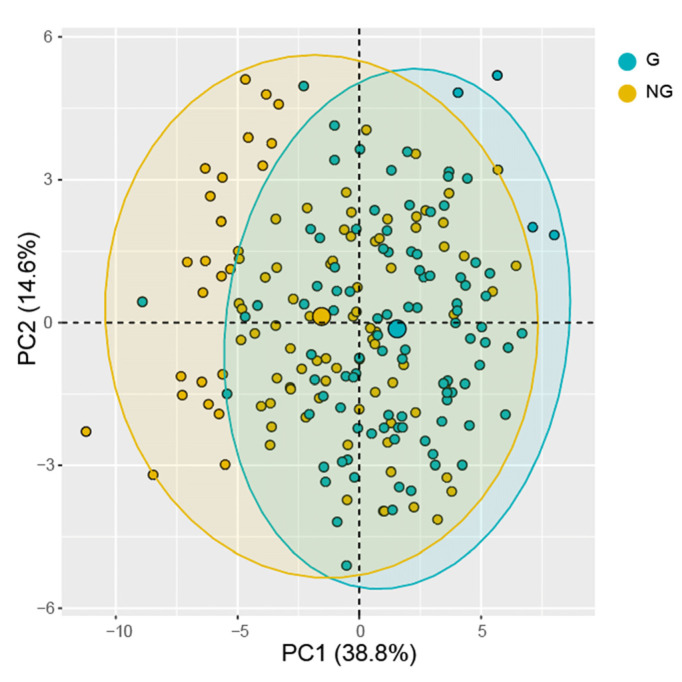
PCA plot based on morphological and multispectral data for germinated and non-germinated seeds.

Furthermore, we tested the performance of multispectral data in predicting seed germination. The five groups of seeds harvested in 2004, 2008, 2011, 2017 and 2019 were divided into two categories: germinating seeds (G) and non-germinating seeds (NG) (Supplementary Figure S6), used for building the discriminant analysis models. The PCA results based on morphological and spectral features showed that the first two principles components explained 62.7% of the original variance (Figure 6). The average accuracy values of SVM and RF model in predicting germinated seeds and non-germinated were 72.0% and 69.7%, respectively. Notably, LDA is also the best model for distinguishing germinated seeds, with high average values of accuracy (97.6%), sensitivity (96.5%) and specificity (98.7%) (Table 2). The top eight important contribution features are all spectral data, such as Band9 and Band11 in LDA, and the morphological features were found in the top eight in SVM and RF (Supplementary Figure S7). 

**Figure 7:**
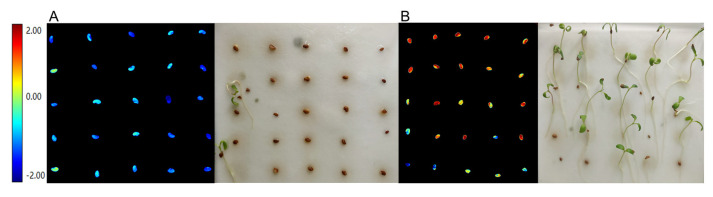
nCDA image vs. actual germination in aged seeds. (**A**), Seeds harvested in 2004 in the nCDA image (**left**) corresponded to their actual germination situation after ten-day imbibition (**right**). (**B**), seeds harvested in 2011 in the nCDA image (**left**) corresponded to their actual germination situation after ten-day imbibition (**right**). The blue seeds in the nCDA image failed to germinate.

We transformed all the spectral images of seeds with colors in nCDA, based on the color standardization of germinated (red) and non-germinated (blue) seeds, and tried to predict the germination or not for a specific seed. The results of nCDA showed that with the increase of storage years, the number of seeds plotted in red color in the nCDA images decreased, while the number of “blue” seeds increased (Table 4). The seeds in 2004 in blue color almost all failed to germinate (Figure 7A), and blue seeds exactly matched the non-germinated seeds, while red ones corresponded to the germinated in 2011 (Figure 7B). Based on the actual seed germination statistics, the average accuracy value of nCDA in predicting germinated and non-germinated seeds ranged from 75.0% to 100.0% (Table 4).

## 4. Discussion

It is promising that smart farming and agriculture employs several types of sensors, such as multispectral imaging, thermal infrared imaging, hyperspectral, and lidar, to increase the quantity and quality of agricultural products with minimal loss and labor [36]. MSI is an emerging, fast, and non-destructive identification method for aged seeds, while it is difficult to distinguish aged seeds and non-aged seeds by visual inspection or traditional experiments. Morphological and spectral features of seeds are related to species, physiological status, substance content, etc., which can be used for seed classification [19]. Previous studies have shown that coat color of the aging seeds became darker gradually with the extension of aging time [25,34]. The traditional visual method to distinguish aged seeds is only based on coat color in the visible light, but the most of multispectral signals and color features (CIELab L*, CIELab A* and CIELab B*) are invisible to the human eye and provide plenty of spectral variations in seed quality [37]. In this study, morphological and spectral features between aged seeds and non-aged seeds were of significant differences. 

In our understanding, the changes of chemical substances in aged seeds result in the reflectance variations of seed. The aged seeds are attributed to the changes in the physical and chemical properties [38]. The malondialdehyde, fatty acid and soluble protein contents increased with an increase of aging time [39], and these changes can be tracked in the visible and near-infrared spectrum [40], as the variations in the visible (375~780 nm) and near-infrared region (780~970 nm) ranges could be contributed by changes of color and physicochemistry in seeds. In cowpea (*Vigna unguiculata*), aged seeds held low reflectivity in visible spectrum and high reflectivity in near-infrared spectrum compared with non-aged seeds [41], which is consistent with the results in this study. Salimi et al. [42] reported that the reflectance showed little differences in the visible spectrum, whereas more variations were found in the near infrared spectrum in a study of processing damage on sugar beet (*Beta vulgaris*) seeds. In contrast, our study showed that alfalfa seeds were distinguishable in near infrared spectrum, and more differences were found in the visible spectrum. This may depend on species and their chemical contents in seeds [23,43,44].

PCA was not able to distinguish aged seeds from non-aged seeds accurately in alfalfa, being consistent with the previous study [31,38]. We notice that the first two principal components in PCA only explained the total variance ranged from 51.0% to 57.8%. In contrast, SVM, RF and LDA achieved high accuracy in distinguishing aged seeds from non-aged seeds, and LDA performed better than RF and SVM in the prediction ability because these prediction models work differently [31,38,45]. The SVM model mainly relies on NIR spectral data in model building, and LDA model mainly relies on visible spectral data, while RF focus on both seed morphologies and spectral indexes (Supplementary Figure S4). The power of multispectral imaging also extended to predict the germination of alfalfa seeds, especially based on nCDA imaging. This nCDA method can produce the transformed color image quickly to determine seed categories in a convenient and high-throughput way of integrating all the spectral data variations [21,24,46].

The seeds from one single batch may be different in qualities, due to the influence of the environment. It is important for seed industry to identify bad quality of seeds in a non-destructive and high-throughput way. Several nondestructive technologies, including multispectral, may be applicable. Near-infrared spectroscopy is rapid and efficient, but its spectrum is not as robust as MSI. Further, X-rays can collect data from interior structures of seeds, but it is costly and radioactive [47]. Hyperspectral imaging can also obtain both spectral and spatial information of seeds, but the data redundancy brings difficulties to data processing [8,9]. Thus, it is necessary to combine MSI and other nondestructive testing techniques for seed testing in the seed industry.

## 5. Conclusions

In this study, multispectral imaging analysis was successfully performed to predict aged seeds harvested in 2004, 2008, 2011 and 2017, in a comparison with those in 2019, and their germination. Multiple spectral and morphological features contributed to the variations between aged and non-aged seeds. The multivariate analysis method of LDA can predict aged seeds and germinated seeds, with the best values of accuracy, sensitivity and specificity. In addition, nCDA can provide transformed spectral images to identify aged and dead alfalfa seeds based on color and spatial information of seeds. In brief, our study clearly shows that multispectral imaging, together with multivariate analysis, is a promising technique in predicting and nondestructive testing of aged and viable alfalfa seeds.

## Figures and Tables

**Table 1:** Morphological features of aged seeds and non-aged seeds.

Feature	2019 (CK)	2004	2008	2011	2017
Area (mm^2^)	2.85 ± 0.42	3.32 ± 0.51 **	3.27 ± 0.58 **	3.25 ± 0.41 **	3.41 ± 0.62 **
Length (mm)	2.39 ± 0.25	2.6 ± 0.24 **	2.62 ± 0.29 **	2.56 ± 0.22 **	2.68 ± 0.27 **
Width (mm)	1.59 ± 0.12	1.69 ± 0.15 **	1.65 ± 0.17 **	1.68 ± 0.12 **	1.7 ± 0.15 **
RatioWidthLength	0.67 ± 0.07	0.65 ± 0.06	0.64 ± 0.06 **	0.66 ± 0.06	0.64 ± 0.05 **
Compactness Circle	0.65 ± 0.07	0.63 ± 0.06 *	0.61 ± 0.07 **	0.64 ± 0.06	0.61 ± 0.05 **
Compactness Ellipse	0.99 ± 0.01	0.99 ± 0.01 *	0.98 ± 0.01 **	0.99 ± 0.01	0.99 ± 0.01 **
BetaShape_a	1.53 ± 0.13	1.5 ± 0.14	1.46 ± 0.14 **	1.51 ± 0.13	1.47 ± 0.11 **
BetaShape_b	1.46 ± 0.14	1.42 ± 0.13	1.39 ± 0.12 **	1.45 ± 0.11	1.4 ± 0.11 **
Vertical Skewness	−0.04 ± 0.03	−0.04 ± 0.03	−0.04 ± 0.03	−0.03 ± 0.03	−0.04 ± 0.03
CIELab L*	48.62 ± 3.39	35 ± 4.37 **	42.94 ± 4.63 **	45.23 ± 4.74 **	44.71 ± 4.91 **
CIELab A*	9.67 ± 1.77	17.21 ± 1.99 **	14.19 ± 2.79 **	13.61 ± 3.18 **	13.01 ± 2.62 **
CIELab B*	31.34 ± 2.46	21.94 ± 4.83 **	28.33 ± 3.42 **	31.34 ± 3.12	29.67 ± 2.87 **
Saturation	32.64 ± 2.21	28.44 ± 4.36 **	31.84 ± 2.71 *	34.27 ± 2.8 **	32.47 ± 2.41
Hue	1.27 ± 0.06	0.91 ± 0.08 **	1.11 ± 0.1 **	1.16 ± 0.1 **	1.16 ± 0.09 *

* and ** indicate significant difference compared with CK at 0.05 and 0.01 levels, respectively, based on independent Student’s *t* test.

**Table 2:** Prediction of aged and germinated seeds based on three multivariate analysis models of SVM, RF and LDA.

Model	Index	2004 vs. 2019	2008 vs. 2019	2011 vs. 2019	2017 vs. 2019	G vs. NG
SVM	Accuracy (%)	99.3	91.3	90.9	87.4	72.0
	Sensitivity (%)	99.6	94.0	93.0	87.6	69.5
	Specificity (%)	99.0	88.8	88.8	87.3	74.4
RF	Accuracy (%)	99.3	89.6	85.5	84.6	69.7
	Sensitivity (%)	99.7	92.3	86.7	85.6	66.5
	Specificity (%)	98.0	86.6	84.3	83.7	72.8
LDA	Accuracy (%)	100.0	100.0	100.0	99.8	97.6
	Sensitivity (%)	100.0	100.0	100.0	99.7	96.5
	Specificity (%)	100.0	100.0	100.0	100.0	98.7

Note: G indicates germinated seeds, and NG for non-germinated seeds. Aged seeds harvested in 2004, 2008, 2011, and 2017 were compared with the non-aged seeds harvested in 2019.

**Table 3:** Contributions of morphological, spectral and “morphological + spectral” data to accuracies in three models of SVM, LDA and RF.

Model	Data	2004 vs. 2019	2008 vs. 2019	2011 vs. 2019	2017 vs. 2019	G vs. NG
LDA	morphological	99.2	87.7	82.2	79.7	71.7
	spectral	99.4	99.4	99.3	98.4	73.1
	morphological+spectral	100	100	100	99.8	97.6
SVM	morphological	99.2	82.8	79.9	76.8	69.0
	spectral	98.8	89.8	87.7	87.3	68.4
	morphological+spectral	99.3	91.3	90.9	87.4	72.0
RF	morphological	98.6	84.8	82.4	76.8	74.5
	spectral	95.1	82.2	78.6	71.6	64.8
	morphological+spectral	99.3	89.6	85.5	84.6	69.7

**Table 4:** The prediction of germination in aged seeds based on nCDA.

Sample	Non-Germinated Seeds	Non-Germinated Seeds Predicted by nCDA	Correctly Predicted Seeds	Accuracy of Predicting Non-Germinated Seeds (%)
2004	95	96	95	99
2008	38	36	29	76
2011	25	19	19	76
2017	20	15	15	75
2019	2	2	2	100

## Data Availability

The morphological and spectral features for five samples with five different storage years and germinated/non-germinated seeds are available at https://figshare.com/s/6d40fb4b879c6c39779f, accessed on 15 June 2021.

## Supplementary Materials

The following are available online at https://www.mdpi.com/article/10.3390/s21175804/s1. Figure S1: Materials of seed; Figure S2: Description of morphological features; Figure S3: Reflectance of 19 wavelengths in all seeds; Figure S4: Relative importance of morphological and spectral features for LDA and SVM models. A, LDA; B, SVM; Figure S5: Relative importance of morphological and spectral features in analysis based on RF model; Figure S6: Morphological features (left) and reflectance of 19 wavelengths (right) in germinated seeds (G) and non-germinated seeds (NG); Figure S7: Relative importance of morphological and spectral features for LDA (left), SVM (middle) and RF (right) models.
