# Supplementary material for: Non-Destructive Identification of Naturally Aged Alfalfa Seeds via Multispectral Imaging Analysis
Source: Sensors (Basel). 2021 Aug 28;21(17):5804. doi: 10.3390/s21175804 (PMC8434479; doi:10.3390/s21175804)
Supplement: Supplementary file 1 [file sensors-21-05804-s001.zip › sensors-1280732-supplementary.pdf]

*sensors*

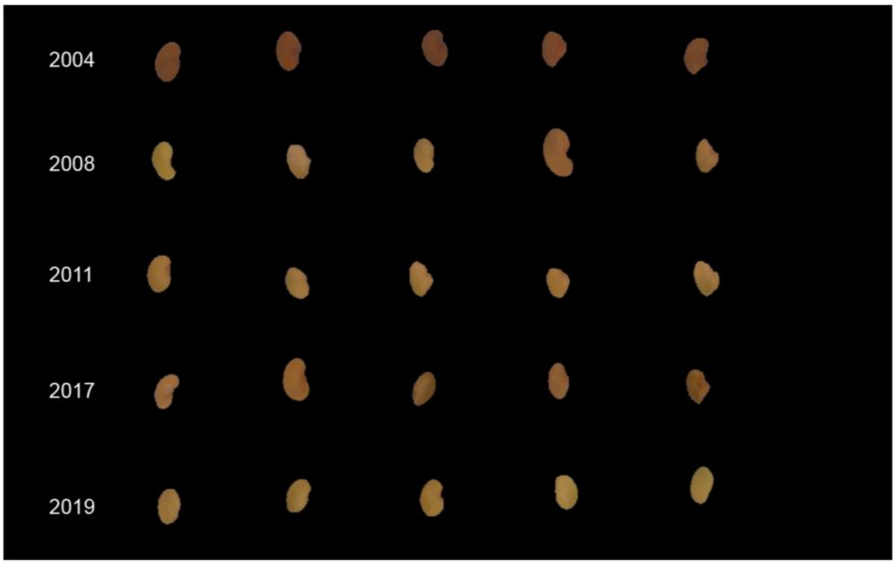


**Figure S1.** Materials of seed.

| Feature | Provider | Description |
|---|---|---|
| Area | Binary Feature | Returns area of blob |
| Length | Binary Feature | Returns length of blob bounding box. |
| Width | Binary Feature | Returns width of blob bounding box |
| RatioWidthLength | Shape Feature | Ratio of width to length of the image oriented bounding box. |
| Compactness Circle | Shape Feature | Compactness of blob defined as 4*Area/(PI*l^2), ratio of object area to the area of a circle with the same length (isolength quotient), length defined by the principle axis of the blob: l=moment1*4. |
| Compactness Ellipse | Shape Feature | Compactness of blob defined as 4*Area/(pi*length*width), ratio of object area to the area of an ellipse with the same length and width (isolength quotient), length defined by the principle axis of the blob: l=moment1*4, and width defined by the secondary axis of the blob: w=moment2*4 |
| BetaShape_a | Shape Feature | Returns parameter a of beta-ellipse fitted to blob mask. Parameter a corresponds to width of most pointed blob-end |
| BetaShape_b | Shape Feature | Returns parameter b of beta-ellipse fitted to blob mask. Parameter a corresponds to width of least pointed blob-end |
| Vertical Skewness | Shape Feature | Skewness of blob mask around horizontal center-axis. |
| CIELab L* | Color Feature | Returns mean Luminance component of CIELab-color of blob |
| CIELab A* | Color Feature | Returns mean A-component of CIELab-color of blob |
| CIELab B* | Color Feature | Returns mean B-component of CIELab-color of blob |
| Saturation | Color Feature | Returns mean saturation of blob based on CIELab coordinates according to formulae: S = SQRT( A^2 + B^2 ) |
| Hue | Color Feature | Returns mean hue of blob based on CIELab coordinates according to formulae: H = ATAN( B/A ) |

**Figure S2.** Description of morphological features.

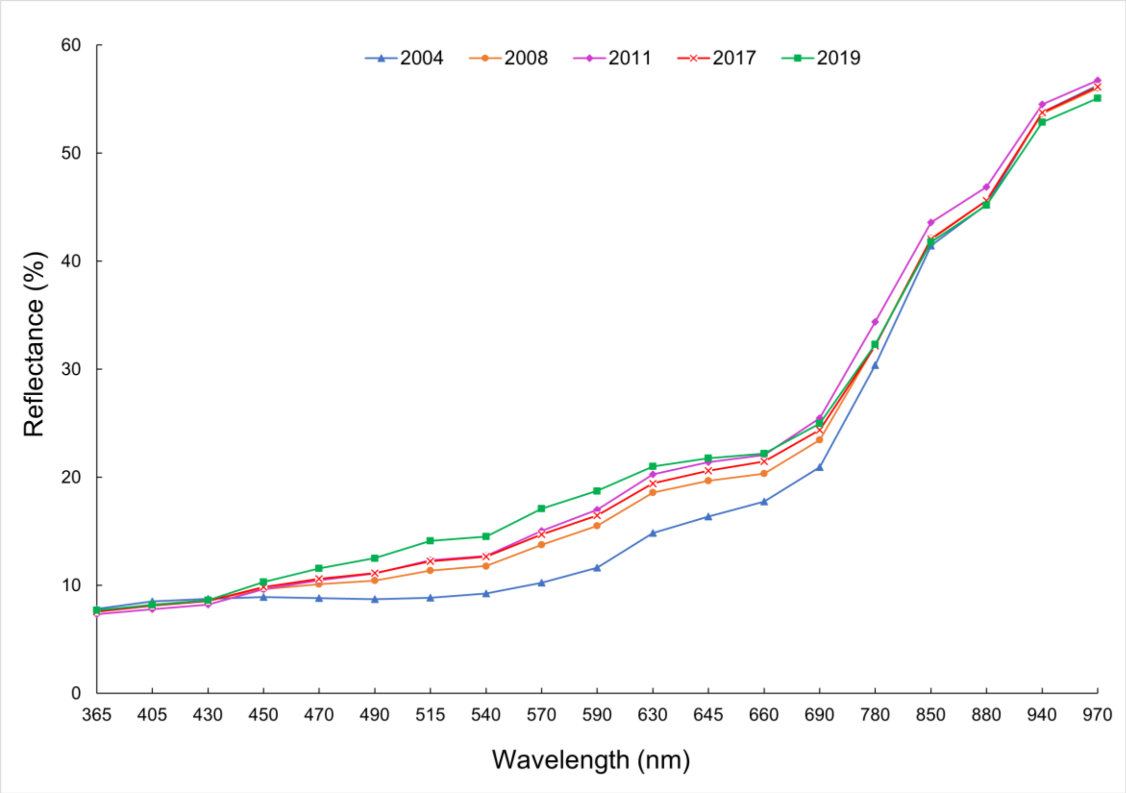


**Figure S3.** Reflectance of 19 wavelengths in all seeds.

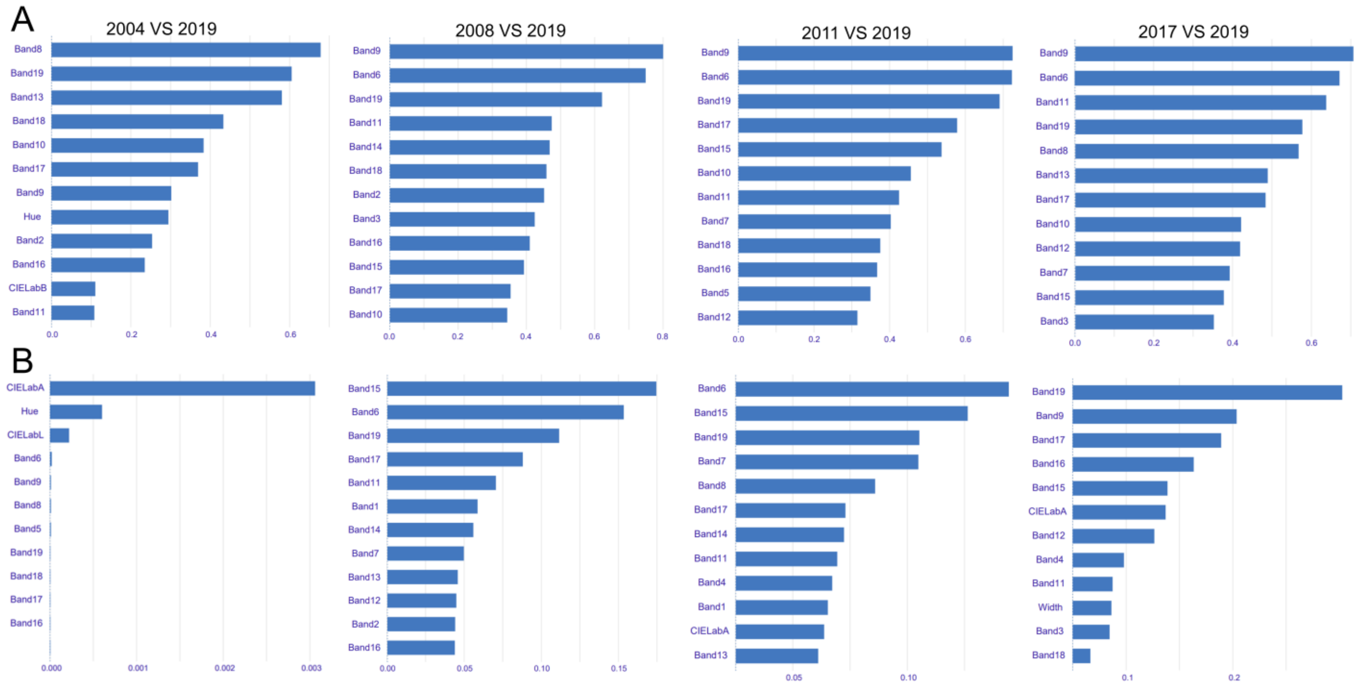


**Figure S4.** Relative importance of morphological and spectral features for LDA and SVM models. A, LDA; B, SVM.

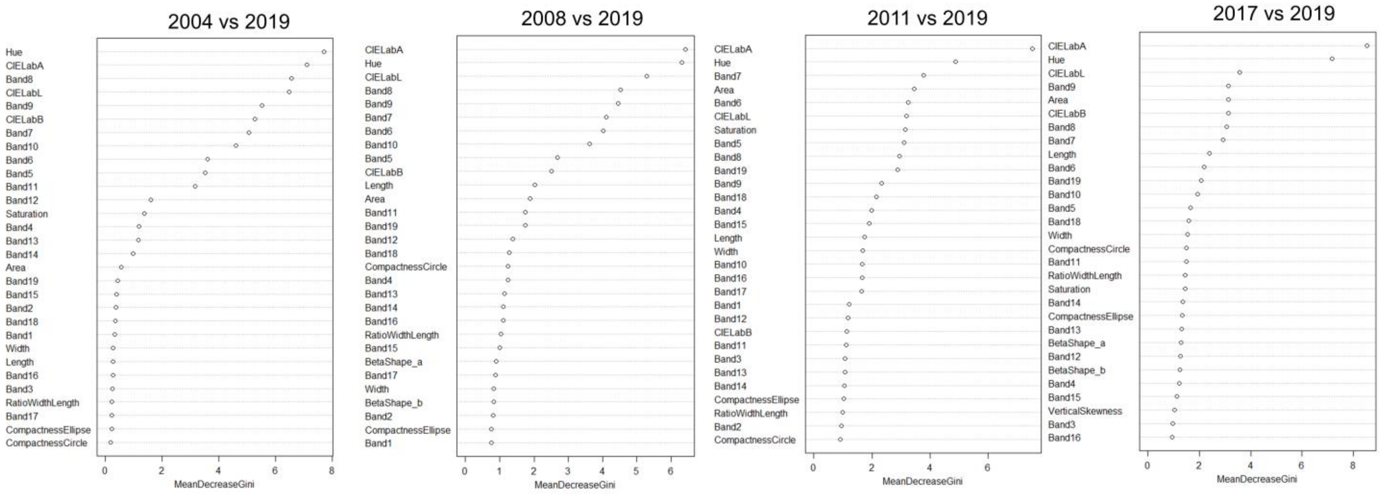


**Figure S5.** Relative importance of morphological and spectral features in analysis based on RF model.

| Feature | NG | G |
|---|---|---|
| Area | 3.27±0.59 | 3.18±0.54 |
| Length | 2.6±0.27 | 2.54±0.28 |
| Width | 1.67±0.16 | 1.66±0.14 |
| RatioWidthLength | 0.65±0.06 | 0.66±0.07 |
| CompactnessCircle | 0.62±0.06 | 0.64±0.08 |
| CompactnessEllipse | 0.99±0.01 | 0.99±0.01 |
| BetaShapea | 1.52±0.14 | 1.5±0.14 |
| BetaShapeb | 1.44±0.14 | 1.43±0.13 |
| VerticalSkewness | -0.04±0.03 | -0.03±0.03** |
| CIELabL | 39.04±6.16 | 46.01±4.24** |
| CIELabA | 14.87±3.36 | 12.66±3.07** |
| CIELabB | 25.76±5.47 | 30.38±2.66** |
| Saturation | 30.35±4.37 | 32.93±2.27** |
| Hue | 1.05±0.15 | 1.18±0.1** |

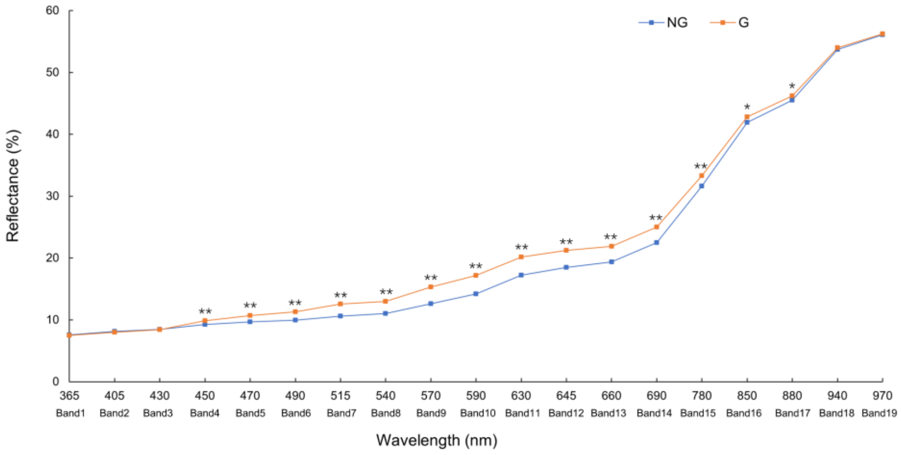


**Figure S6.** Morphological features (**left**) and reflectance of 19 wavelengths (**right**) in germinated seeds (G) and non-germinated seeds (NG).

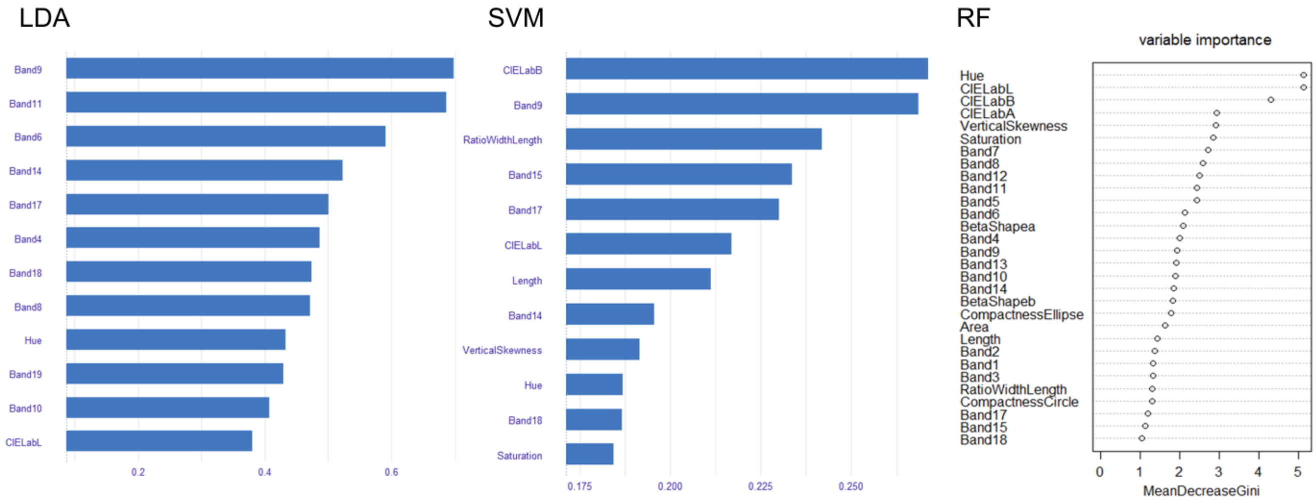


**Figure S7.** Relative importance of morphological and spectral features for LDA (**left**), SVM (**middle**) and RF (**right**) models.
